# Parental Attitudinal and Behavioral Change Associated With Prevention-Focused Parenting Education: An Interpretive Description

**DOI:** 10.1177/10901981211033233

**Published:** 2021-10-04

**Authors:** Karen M. Benzies, Jana Kurilova, Mathilde van der Merwe

**Affiliations:** 1 University of Calgary, Calgary, Alberta, Canada

**Keywords:** infants, interpretive description, mechanisms of change, parenting education, preschool children, preventive programs

## Abstract

Prevention-focused parenting education programs (P-FPEPs) provide knowledge and support to parents to strengthen parent–child relationships, enhance parental and family well-being, and promote healthy child development. The positive impact of such programs on child health and development is well documented. Yet, how P-FPEPs influence parents remains unclear. The objective of this study was to explore parental perceptions of changes associated with participation in a P-FPEP. We analyzed data using interpretive description with qualitative responses from 459 parents who participated in nine different P-FPEPs in a large Canadian city. Participation in a P-FPEP changed parents’ relationships with themselves, their children, their partners, and their community. Participants’ relationship with themselves as parents changed as they recognized the value of self-care without guilt, gained knowledge of typical child development, and developed greater confidence in their parenting. Positive changes in participants’ relationships with their children were facilitated by better understanding the perspective of the child, improving communication, feeling more connected to their child, and changing parenting behavior. For many participants, the relationship with their partner improved when they learned about different parenting styles and began communicating more openly. Participants’ relationships with the larger community were strengthened as they experienced a sense of normalization of their parenting experiences, developed connections with other parents, and learned about community resources. Independent of any specific program curriculum or structure, change associated with P-FPEPs focused on how a shift in understanding and attitudes changed relationships and consequently changed parenting behavior.

Parents are most influential in the earliest years when their child’s brain is developing rapidly and nearly all experiences are shaped by the family environment (Shonkoff & Phillips, 2000). High-quality parent–child relationships enhance child health and well-being (Kaminski et al., 2008; O’Connor, 2002; Shonkoff, 2003). The term “parenting” has been used interchangeably with parental care, parental functioning, and parent–child relationship (e.g., Belsky, 1984; O’Connor, 2002), and was described by Belsky (1984) as a system where three major determinants interplay: personality/psychological well-being of the parent, characteristics of the child, and contextual sources of stress/support. Shonkoff and Phillips (2000) described parenting as the relationship between a young child and the adult(s) most emotionally invested in and available to them. Based on these definitions, one can think of parenting as the parent–child relationship in context.

Parent–child relationships support early childhood development (Shonkoff & Phillips, 2000). Parents who are more knowledgeable about (a) what to expect, (b) effective ways to respond, and (c) when to be concerned about their children are more likely to be able to support their children’s development (Morris et al., 2018; National Academies of Sciences, Engineering, and Medicine [NASEM], 2016). Yet, many parents have limited knowledge of typical child development, particularly in the domains of social and emotional development (Bornstein et al., 2010; Rikhy et al., 2010), and can benefit from information and support (Morris et al., 2018; NASEM, 2016). Such information and support can be successfully imparted through parenting education programs—interventions designed to enhance parental role performance through training (Deković et al., 2012). Parenting education has a positive influence on parents and their children (Barlow et al., 2014; Barlow et al., 2016; Deković et al., 2012). However, most parents do not seek assistance until their child is having serious difficulties and requires time-consuming and expensive clinical interventions (Axford et al., 2012). Prevention-focused parenting education programs (P-FPEPs) are distinct from treatment programs in that they seek to increase family resilience and parental competency in populations that are not in crisis, with an aim to prevent problems before they occur and minimize the need for clinical interventions (Ştefan & Miclea, 2010; Wakschlag et al., 2019). We use the term P-FPEP to describe programs that provide education and support at either the primary or the secondary prevention level (Wakschlag et al., 2019). The goal of such programs is to (a) promote healthy child development, (b) strengthen parent–child relationships, and (c) enhance parental and family well-being.

P-FPEPs have demonstrated short- and long-term improvements in parenting knowledge and skill (Sandler et al., 2011; Sandler et al., 2015). In contrast to behavior-based parenting programs (e.g., 1-2-3 Magic [www.123magic.com], Triple P [www.triplep.net]), P-FPEPs focus on communication as the foundation of healthy relationships between children and their parents (Morris et al., 2018; Ştefan & Miclea, 2010). P-FPEPs recognize that children’s cognitive, social, and emotional development happens in the context of healthy and sustaining parent–child relationships (Morris et al., 2018; Ştefan & Miclea, 2010). Such programs can be considered effective if they develop parental understanding of the interactional nature of the parent–child relationship (Miller & Sambell, 2003).

While there has been significant research on improved child outcomes resulting from P-FPEP, much less attention has been paid to the changes that occur within parents themselves (Teti & Cole, 2011). The process model of parenting developed by Belsky (1984) suggests that the personal psychological resources of a parent have the greatest potential for protecting optimal parental functioning. Yet, how parenting programs exert their effects on parents still appears to be a “black box.” Despite curricula and logic models that outline expected changes in parenting knowledge and behaviors, there is a lack of research on how parents apply learning to change behavior. The purpose of this study was to explore parental perspectives of change associated with participation in P-FPEPs.

## Potential Theories Underlying P-FPEPs

Several theories have been used to explain the influences of P-FPEPs on parental attitudes and behaviors. First, adult learning theory posits that participants bring their past knowledge and experiences to new learning (Jarvis, 1987; Mezirow, 1997) and that they need knowledge and support that are relevant and timely (Knowles et al., 2012). Parents may need time to reflect on their own parenting before they can apply new knowledge (Schön, 1987). Second, Vygotsky (1978) posits that knowledge is socially constructed and therefore learning is a collaborative effort where one establishes and maintains knowledge among a community of knowledgeable peers. Finally, the theory of planned behavior suggests that attitudes, social norms, and perceived behavioral control predict intended behaviors, which in turn predicts actual behaviors (Ajzen, 2011). These principles surely apply to P-FPEPs, yet there is a lack of research about the actual mechanisms of change and potential theoretical underpinnings. Mechanisms of change can be defined as learning and value shifts that can be cognitive, attitudinal, emotional, or mental (Ghate, 2018). Such shifts facilitate or mediate the outcome, which in P-FPEPs includes behavior change in parents (Ghate, 2018).

## Methods

### Design and Setting

Using interpretive description (Thorne, 2013), we conducted a constructionist inquiry of qualitative data collected as part of a larger study to assess the psychometric properties of the UpStart Parent Survey (Benzies, Barker, et al., 2013). We collected data for the larger study between April 2010 and June 2012 in a western Canadian city (population 1 million). Increasing emphasis on the effects of parenting on lifelong learning and health (NASEM, 2016; Sandler et al., 2015; Wakschlag et al., 2019) prompted this secondary analysis. The Mount Royal University (ID 2010-43) and University of Calgary (ID 23642) ethics boards approved the study. We applied the Consolidated Criteria for Reporting Qualitative Research to this article (Tong et al., 2007).

### Participants

P-FPEPs were included if they demonstrated capacity to engage in the study and wanted an individualized program evaluation report. The participants in the larger study were a convenience sample of 536 parents of infants and young children who had attended a P-FPEP and completed the UpStart Parent Survey (Benzies, Barker, et al., 2013). Participants from a cross-cultural parenting program (*n* = 36) that included international nannies were excluded because their responses sometimes referred to their own children back home and sometimes to the children they were caring for in Canada. Of the remaining 500 participants, 459 contributed qualitative data and were included in this study. See Table 1 for participant characteristics. The study participants were similar to the population in marital status, education, income, and ethnicity (City of Calgary, 2017).

**Table 1.** Participant Characteristics (*N* = 459).

Characteristic	*n*	%
Gender (*n* = 441)		
Female	390	88.4
Male	51	11.6
Age (years, *n* = 444)		
Under 18	2	0.5
18-29	121	27.3
30-39	258	58.1
40-49	53	11.9
50 or over	10	2.3
Marital status (*n* = 443)		
Single	45	10.2
Married/common law	385	86.9
Divorced/separated	13	2.9
Highest education level (*n* = 442)		
Less than high school	56	12.7
High school	52	11.8
Certificate/diploma program	59	13.3
College/university degree	275	62.2
Annual household income ($, *n* = 415)		
<20,000	76	18.3
20,000–40,000	43	10.4
40,000–80,000	80	19.3
>80,000	216	52.0
Ethnicity (*n* = 418)		
Caucasian/White	310	74.2
Indigenous	32	7.7
East or Southeast Asian	39	9.3
Latin American	9	2.2
Other^a^	28	6.7
Born in Canada^b^ (*n* = 439)	362	82.5
First parenting program attended^b^ (*n* = 441)	276	62.6
Number of children in household (*n* = 426)		
0	39	9.2
1	186	43.7
≥2	201	47.2

*Note.* The value of *n* varies due to missing data.

a Categories with *n* ≤ 8; includes South Asian, Black, Arab/West Asian, and other. ^b^Reflects the number and percentage of participants answering “yes” to this question.

### Prevention-Focused Parenting Education Programs

Six community agencies offering nine different P-FPEPs targeting different subpopulations of parents of children from birth to 6 years participated in the study (see Table 2). The programs lasted between 4 and 13 weeks; the classes lasted between 1.5 and 3 hours. Well-established curricula focused on typical child growth and development, parenting strategies, and community resources. Some programs included only classroom learning/discussion, whereas others included classroom learning/discussion and components where parents and children learned together.

**Table 2.** Description of Included Prevention-Focused Parenting Programs (in Alphabetical Order).

Program	Program description
Baby and Me	Twelve weekly 2-hour, fully subsidized sessions that target parents of infants under 1 year. Baby and Me is facilitated by public health nurses. Parents learn about infant care, make new friends, and have time to ask parenting- and health-related questions. The format is relaxed and inviting, with parents and babies playing and learning together.
Baby and You for Moms (Calgary Health Region, 2007)	Four weekly 2-hour, fully subsidized sessions that target first-time mothers of infants between 2 and 9 months. Baby and You is facilitated by public health nurses. Parents connect with other parents and learn about infant care and development (including brain development), parenting, stress, attachment, and community resources.
Literacy, Engagement, Attachment, Play	Eight weekly 2-hour, fully subsidized sessions that include structured and unstructured play time. During the structured time, parents and children are introduced to rhymes, songs, and oral stories designed to connect parents with their children, enhance the child’s oral language, and encourage development. Participants receive education on the developmental and behavioral stages of children, learn about community resources, and can address any parenting concerns in an open forum.
Make the Connection (The Psychology Foundation of Canada, 2003)	Eight weekly 2-hour, fully subsidized sessions for parents of children from birth to 12 months. Make the Connection trained facilitators focus discussion on what infants need for a good start in life, including traditional customs, attachment, and play activities to promote positive parent–infant relationships and secure attachment. Make the Connection gives parents the opportunity to engage in conversations on how they feel about parenting young children.
Nobody’s Perfect (Public Health Agency of Canada, 2016; nobodysperfect.ca)	Six 2- to 3-hour fully subsidized sessions over 1-3 weeks that target the expressed learning needs of parents who are young, single, and socially or geographically isolated, and have low income and/or limited formal education. Facilitated by a certified parenting educator, Nobody’s Perfect helps parents of children from birth to age 5 years to (a) understand children’s health, safety, and behavior; (b) build on the skills they have and learn new ones; (c) improve self-esteem and coping skills; (d) increase self-help behaviors; (e) provide support to one another; and (f) connect with community services and resources.
Parent Effectiveness Training (Gordon, 1970)	Eight 3-hour sessions facilitated by certified instructors at a cost of $195CDN that target parents of children 2 years and older. Parent Effectiveness Training helps parents (a) learn skills to enhance parent–child relationships, (b) talk to their children so that they will listen, (c) listen to their children so they feel genuinely understood, (d) resolve conflicts and problems in the family, and (e) troubleshoot family problems and know which skills to use to solve them.
Parents and Children Together (www.pactcalgary.com)	Eleven weekly 2-hour sessions at a cost of $180CDN that target parents of children from birth to 5 years to enhance parental capacity by advancing parenting knowledge and skills as well as furthering healthy parental attitudes and values. Facilitated by a parenting educator, parents alternate each week between a parenting education discussion group and a play-focused activity with the children. All parents join in the final 15 minutes of song and story time. Discussion groups cover topics such as stages of development, parenting styles, effective discipline, communication, and fostering self-esteem.
Terrific Toddlers^a^ (Alberta Health Services, 2008)	Five weekly 2-hour sessions that target parents of children 1–4 years old. Terrific Toddlers helps parents to (a) understand child development in the toddler years; (b) understand the impact development has on their child’s health, safety, and behavior; (c) increase confidence in their ability to cope with the challenges of living with a toddler; (d) understand growing autonomy as a necessary developmental stage; (e) access mutual parental support; and (f) gain increased awareness of community resources.
TLC Parenting for Toddlers	Thirteen weekly 1.5-hour drop-in, fully subsidized sessions for parents of children 12–36 months old. TLC Parenting for Toddlers helps parents learn about child development, sleep, nutrition, literacy, crafts, and play from expert presenters. Parents are encouraged to connect with one another and community resources.

*Note.* References are not available for Baby and Me; Literacy, Engagement, Attachment, Play; and TLC Parenting for Toddlers.

a Terrific Toddlers was offered by two agencies; one agency fully subsidized the program, while the other charged $120CAD.

### Data Collection

We collected data using the UpStart Parent Survey, a reliable and valid, self-report, retrospective pretest–posttest measure of common outcomes expected of P-FPEPs (Benzies, Clarke, et al., 2013). The survey includes three scales: (a) Parenting Knowledge/Skills (10 items), (b) Parenting Experience (11 items), and (c) Program Satisfaction (seven items); and it has three text boxes for written responses to the following open-ended questions: (a) What are three things I learned from this program? (b) What are two things I have done differently because of this program? and (c) What is one thing I still have questions about? The survey takes about 15 minutes to complete. The responses to the first two open-ended questions constituted the data for this study.

### Procedures

At the end of the last session, the program facilitator used a script to invite the participants to complete the survey. The facilitator remained in the room to answer questions and collect the surveys. Consent to participate was indicated by returning a completed survey. The completed surveys were posted to the research office in a stamped, self-addressed envelope. The responses to the open-ended questions were transcribed verbatim and managed in MS Excel.

### Data Analysis

Data analysis occurred after the data collection was completed. Two coders inductively analyzed the data thematically according to Braun and Clarke (2006). First, the coders independently reviewed the participant responses to the open-ended questions from the surveys to familiarize themselves with the data. Second, they identified ideas and coded data by applying labels to the emerging ideas. Third, in two in-person meetings, the coders identified patterns in the codes, then defined and named the main themes. During these meetings, the two coders achieved consensus through discussion. Throughout the data analysis, the coders considered how the potential theories informed the emerging themes. The final themes were validated with the executive directors and program facilitators of four agencies.

## Findings

The participants reported that P-FPEPs shifted their knowledge and attitudes about their role as a parent, which changed their relationships with themselves, their children, their partners, and the community and reportedly changed their parenting behavior. Quotes are attributed by participant ID and program. One participant, who captured the essence of the changes related to P-FPEPs, wrote that “the ‘relationship’ is the most important thing” (0098 Terrific Toddlers). The themes (relationships with self, child, partner, and community) and subthemes are described below.

### Relationship With Self

#### Self-Care Without Guilt

The participants learned that their personal health and well-being were critical to the health and well-being of their children. One mother wrote that she needed to “take care of myself so that I can take care of my family” (0125 Baby and Me). Another realized that she needed to shed the guilt associated with self-care: “I have learned to look after myself without feeling guilty” (0028 Parents and Children Together [PACT]). With better self-care, another participant “learned to face my problems head on and deal with them in a positive, productive, healthy way” (2552 Nobody’s Perfect). Participants internalized the knowledge they gained through self-awareness and reflexivity about their behaviors and thought processes. One mother decided to “not make decisions based on my ego but what is best for my kids” (0085 Terrific Toddlers).

#### Knowledge

Information about child development was the core content in most P-FPEPs. Some participants articulated a clear understanding of developmental milestones, such as “My child is not yet at the milestone where she understands sharing” (0052 PACT). Greater understanding of milestones created more reasonable expectations of child behavior. One mother wrote, “[I] found myself less frustrated because I have learned that this is just what kids do at different stages” (0007 PACT). Another wrote that she has “been more patient because I understand her development” (0664 PACT). Similarly, another wrote that she “relaxed more in watching children play (let them be kids)” (2509 PACT). As knowledge of developmental milestones increased, parenting behaviors changed. One mother wrote, “[I] started having [my] baby sit at the dinner table with us instead of putting him somewhere else” (0176 Baby and You). Another wrote that she learned, “Talk, read and listen to my baby is important to [my] baby’s development” (0247 Make the Connection).

Increased knowledge of developmental milestones normalized aspects of children’s behaviors that were concerning to parents. A mother of a toddler learned that “temper tantrums are part of normal development and how to deal with them” (0025 PACT). Normalization enabled participants to realign “our expectations of our son (more realistic for his age)” (0096 Terrific Toddlers). “Just knowing that many issues are ‘normal’ and you will survive them” (1004 PACT) was reassuring.

#### Confidence

With increased knowledge, the participants gained confidence in their parenting abilities. One learned “to have confidence in myself as a parent” (2258 Nobody’s Perfect). Another wrote that she gained “more confidence in parenting when you understand your child’s developmental stage and perspective” (0031 PACT). One participant felt reassured “that I’ve been doing lots of things ‘right’” (0021 PACT). Another wrote, “My confidence as a parent has improved, and I feel so much more capable of enjoying motherhood because of the tools learned” (0124 Baby and Me).

### Relationship With Their Child

#### Understanding the Perspective of the Child

Understanding the perspective of the child was key for many participants. One participant wrote that she learned “to respect my children as I would like to be respected” (0084 Terrific Toddlers). With a better understanding of the child’s point of view, another wrote, “I try to look for the need behind the behavior” (0121 Parent Effectiveness Training). Simple strategies contributed to understanding the child’s perspective. One participant wrote that she “learnt to get down eye to eye and view what I am doing more from the child’s perspective” (0087 Terrific Toddlers).

#### Communicating

After participation in a P-FPEP with an observation/practice component, one mother wrote that she was “listening more to what my children are saying or what they are upset about/acknowledging their feelings” (0064 PACT). Another wrote, “I have taken more time to hear what my daughter is ‘saying’” (0244 Make the Connection). Linked to changes in the relationship with self, one participant wrote about the importance of “handling my emotions *not* with yelling—using ‘I’ statements to communicate” (0070 PACT). Participants learned practical communication tools, such as “using when/then statements” (1015 PACT), which reportedly changed their behaviors in interactions with their children.

#### More Connected to the Child

One participant wrote about being “more connected to [the] child” (0004 PACT). Another wrote about strategies such as “eye contact to make connection with [my] baby” (0246 Make the Connection). Yet another wrote that she “spent more time with my kids because I appreciate them more” (0050 PACT). Changes in perceptions about the importance of connections with their child transferred to relationships with others. One participant wrote that she “made more time to connect with all family members in a more direct way” (0653 PACT).

#### Changing Parenting Behavior

Participants reported that they changed their parenting behaviors. One wrote,

I do not spank [or] use time-outs. Instead of always saying “no,” I say, “Yes, you can after dinner (etc.).” I play with my child and read stories and realize this is how she learns. I tell her I’m proud of her . . . when her behavior allows us to get grocery shopping (etc.) done. (0013 PACT)

One participant wrote, “I have not yelled at my son; I am more patient with him and more understanding” (0213 Nobody’s Perfect). Another wrote, “I have consciously spent more time and put . . . more effort into playing with, talking to, and reading to my baby—not just taking care of her” (0307 Baby and You). A participant with a preschooler wrote that she now “problem solved with my children, instead of *for* them” (2033 PACT).

### Relationship With Partner

#### Respect for Different Parenting Styles

Participants learned about different parenting styles, which provided perspective and respect for their partner’s approach to parenting. One participant wrote that she learned, “It’s ok to have different parenting styles” (007 PACT). Another wrote that she learned how to “respect how your partner parents—it’s their relationship with the child, and they own it” (0016 PACT).

#### Opened Communication

Increased knowledge of parenting styles “opened communication with my spouse on how each of [us] approaches parenting” (0016 PACT). One participant “initiated more conversation with regards to parenting [and] tried/trying to implement parenting strategies proactively over ‘fly by the seat of my pants’” (2510 PACT). A participant in a program for parents who were separating wrote that “partners do not have to agree and need to follow through on their own actions” (0101 Parent Effectiveness Training).

### Relationship With Community

#### Normalizing Parenting

One participant wrote, “I learned that I was not alone in the way I felt and things I didn’t know” (0130 Baby and Me). Another wrote, “We are normal and dealing with what everyone is dealing with” (0087 Terrific Toddlers). The realization “that other mothers go through similar issues and are supportive rather than judgmental” (0021 PACT) was reassuring.

#### Improved Connection With Other Moms

One participant realized, “I can get support from other moms” (0074 PACT). Another wrote, “There are lots of other moms that share my joy, questions, and horrors about parenting”; she added, “It’s fun to share the happy aspects of motherhood and reassuring to talk about our worries” (0065 PACT). Engaging with other mothers reduced social isolation. As one participant put it, “I stayed home all the time, and now I get out and meet a lot of moms that I can be friends with” (0022 PACT).

#### Increased Awareness of Community Resources

One participant wrote that she “encouraged [her]self to understand and accept being a young parent—it’s ok to ask for help” (2544 Nobody’s Perfect). Another wrote, “Parents are human too; we can ask some organization for help” (0218 Nobody’s Perfect). Participants learned about “how to find the support I need when I need it” (2558 Nobody’s Perfect), including “resources at the library and in the city” (0299 Baby and You).

## Discussion

In this study, we identified parental perspectives of change associated with participation in nine different P-FPEPs offered by six different agencies. P-FPEPs changed the participants’ knowledge and attitudes about their role as a parent, which changed their relationships with themselves, their children, their partners, and the community and reportedly changed their parenting behavior.

As parents developed an appreciation for self-care without guilt, gained a deeper understanding of their child’s developmental stage, believed that their concerns were normalized, and developed greater confidence in their parenting skills, they changed their attitudes about their role. Recognition of the importance of self-care is a novel finding in this study. For many participants, engaging in self-care activities without feeling guilty represented a profound shift in their relationship with self, which may represent a psychological resource important for parenting (Belsky, 1984). In a synthesis of four qualitative studies of treatment-focused parenting programs, Kane and colleagues (2007) identified a reduction in self-blame and guilt associated with loss of control in parenting behaviors. Our study may be the first to suggest that P-FPEPs reduced guilt related to parental self-care.

Consistent with the literature (NASEM, 2016), with increased knowledge of developmental milestones, the participants became more patient and understanding of their child’s behavior, and some realized that they needed to reevaluate their expectations. Increased knowledge reassured participants that their children’s behaviors were typical (NASEM, 2016). Participants commented on increased confidence in their parenting skills and abilities. Increased confidence may lead parents to believe that they can improve their parenting skills, which may subsequently lead to behavior change. This is a common finding in qualitative studies of experiences in parenting programs—both prevention (Mejia et al., 2016; Patterson et al., 2005; Wolfe & Haddy, 2001) and treatment (Furlong & McGilloway, 2012; Kane et al., 2007) focused.

Consistent with a systematic review and meta-synthesis of qualitative literature that identified developing a better understanding of and an improved relationship with the child as a subtheme in the family’s journey through a parenting program (Butler et al., 2020), the participants in this study described numerous examples of positive changes in their relationship with their child. This was facilitated by understanding the perspective of the child, improved communication, changes in parenting behavior, and feeling more connected to their child. The positive changes in the parent–child relationship allowed parents to appreciate their children for who they are and to truly enjoy spending time with them. Patterson et al. (2005) described a similar experience of parents’ relationships with their children becoming much more rewarding during their P-FPEP. Analogous to our findings, much of the literature has pointed to learning more effective ways to communicate and implementing new parenting skills and behaviors as essential components of parenting programs (Kaminski et al., 2008; Sandler et al., 2011; Sandler et al., 2015).

A better understanding of the child’s perspective, to get to their level and see things from their point of view, is a less commonly reported finding; however, this was perceived as key for many parents in this study and also highlighted by Wolfe and Haddy (2001). The concept of perspective taking as a component of reflective functioning was first defined in the context of parent–child relationships by Fonagy and colleagues (1991) as the “capacity to reflect upon mental functioning in oneself and others” (p. 205). Even though it is now almost three decades since Fonagy et al. (1991) identified parental reflective functioning as critical to the development of healthy parent–infant relationships and more than a decade since the first reflective parenting interventions were developed (Slade, 2007), recent years have seen renewed and more widespread interest in the topic and its clinical applications (Luyten et al., 2017). The findings of our study suggest that increased parental reflective functioning may play a role in the mechanisms through which P-FPEPs enhance parent–child relationships.

The changes in the participants’ relationships with themselves and their children extended to their relationships with other family members and the community. Through learning about different parenting styles, participants developed understanding and respect for their partner’s approach to parenting. Several participants commented on having more open communication with their partner. The findings related to the relationship with partner are novel in the P-FPEP literature. In contrast, concepts related to the relationship with the community are commonly reported. Participants in this study commented on how P-FPEPs helped them nurture supportive relationships with other parents. Nonjudgmental support of other group members has been identified as particularly beneficial by parents in prevention-focused (Patterson et al., 2005; Wolfe & Haddy, 2001) as well as treatment-focused (Furlong & McGilloway, 2012; Kane et al., 2007) programs. The Building Early Relationships Model of Change (Morris et al., 2017) emphasizes the important role of P-FPEPs in promoting nurturing and supportive relationships not only between parents and children but also between parents and other adults. Morris et al. (2017) proposed that promoting such relationships can positively affect parent and child well-being through the mechanisms of strengthening parental social support networks and increasing positive parent–child interactions.

In agreement with Belsky’s (1984) suggestion of the role of personal psychological resources in optimal parental functioning, our study confirmed several such resources that contribute to behavior and relationship change. Self-care and confidence were resources for changes in the relationship with self, and perspective taking as a component of reflective functioning was a resource for changes in the relationship with the child. The mechanisms of change at play here can be viewed as learning and value shifts of a cognitive, attitudinal, emotional, or mental nature (Ghate, 2018) that can mediate behavior change in parents. It appears that these learning and value shifts were also transferrable to relationships with the participant’s partner and the community.

### Limitations

This study was strengthened by the large sample that provided data from different P-FPEPs. The study was limited by the secondary analysis of parental responses to open-ended questions that were part of a program evaluation survey administered at the end of a P-FPEP. The participants who completed the survey were positive about their experiences, which may be different for those who did not complete the program or the survey. There is no information available about participant dropout; thus, caution is warranted regarding transferability of these findings to other P-FPEPs. As with many studies of parenting programs, most of the participants were mothers; future research is needed to capture fathers’ perspectives. Finally, there were six different agencies offering nine different parenting programs that focused on different populations and child age-groups. The subthemes identified may therefore not apply to all the programs in the study.

### Implications for Future P-FPEP Design

Independent of any specific program curriculum or structure, change associated with P-FPEPs focused on how a shift in attitudes and knowledge changed relationships and consequently changed parenting behavior. This suggests that P-FPEPs should focus education and support on helping parents understand the mechanics of relationships. Such mechanics include consideration of the perspective of the other in interactions, as well as how the interactive space can be used to recognize and respond appropriately to children’s social cues. P-FPEPs can lead to changed relationships if they are designed around four theoretical pillars. Relational communication (Benzies, 2016), which recognizes communication as the foundation of healthy parent–child relationships, should form the first pillar. Careful attention to adult learning, with reflection and timely and accessible parenting education and strategies, should serve as the second pillar. Given that learning takes place in a community of peers, intentionally designed opportunities for supportive interactions with children and other parents should form the third pillar. Embedding strategies that teach parents how to implement and sustain change over time is critical. Such strategies should focus on changing parental attitudes, social norms, and perceived behavioral control as foundational to changing intentions, and ultimately changing behaviors. Thus, the theory of planned behavior (Ajzen, 2011) forms the final pillar. Designing and evaluating P-FPEPs with these theories in mind may improve parental experiences and child and family well-being.
